# Toxicity and Functional Impairment in Human Adipose Tissue-Derived Stromal Cells (hASCs) Following Long-Term Exposure to Very Small Iron Oxide Particles (VSOPs)

**DOI:** 10.3390/nano10040741

**Published:** 2020-04-13

**Authors:** Katrin Radeloff, Andreas Radeloff, Mario Ramos Tirado, Agmal Scherzad, Rudolf Hagen, Norbert H. Kleinsasser, Stephan Hackenberg

**Affiliations:** 1 Department of Otorhinolaryngology, Head and Neck Surgery, Evangelisches Krankenhaus, Carl von Ossietzky-University, 26129 Oldenburg, Germany; andreas.radeloff@uni-oldenburg.de; 2 Department of Otorhinolaryngology, Plastic, Aesthetic and Reconstructive Head and Neck Surgery, Julius-Maximilian-University, 97070 Würzburg, Germany; mramos76@yahoo.com (M.R.T.); scherzad_a@ukw.de (A.S.); hagen_r@ukw.de (R.H.); hackenberg_s@ukw.de (S.H.); 3 Department of Otorhinolaryngology, Head and Neck Surgery, Kepler-University, 4040 Linz, Austria; Norbert.Kleinsasser@kepleruniklinikum.at

**Keywords:** iron oxide nanoparticles, VSOP, nanoparticles, toxicity, differentiation potential, human adipose-derived stromal cells, stem cells, long-term exposure

## Abstract

Magnetic nanoparticles (NPs), such as very small iron oxide NPs (VSOPs) can be used for targeted drug delivery, cancer treatment or tissue engineering. Another important field of application is the labelling of mesenchymal stem cells to allow in vivo tracking and visualization of transplanted cells using magnetic resonance imaging (MRI). For these NPs, however, various toxic effects, as well as functional impairment of the exposed cells, are described. The present study evaluates the influence of VSOPs on the multilineage differentiation ability and cytokine secretion of human adipose tissue derived stromal cells (hASCs) after long-term exposure. Human ASCs were labelled with VSOPs, and the efficacy of the labelling was documented over 4 weeks in vitro cultivation of the labelled cells. Unlabelled hASCs served as negative controls. Four weeks after labelling, adipogenic and osteogenic differentiation was histologically evaluated and quantified by polymerase chain reaction (PCR). Changes in gene expression of IL-6, IL-8, VEGF and caspase 3 were determined over 4 weeks. Four weeks after the labelling procedure, labelled and unlabelled hASCs did not differ in the gene expression of IL-6, IL-8, VEGF and caspase 3. Furthermore, the labelling procedure had no influence on the multidifferentiation ability of hASC. The percentage of labelled cells decreased during in vitro expansion over 4 weeks. Labelling with VSOPs and long-term intracellular disposition probably have no influence on the physiological functions of hASCs. This could be important for the future in vivo use of iron oxide NPs.

## 1. Introduction

The labelling of cells, e.g., stromal cells (ASCs) derived from adipose tissue, with iron oxide nanoparticles (IONPs) is a frequently used tool in the field of regenerative medicine and biomedical research [1]. Non-invasive visualization and long-term in vivo tracking by means of magnetic resonance imaging (MRI) allows researchers and practitioners to determine the effectiveness and success of stem cell treatment after cell transplantation [2,3]. Furthermore, IONPs are used in tissue engineering [4], in targeted drug and gene therapy or as tools for cancer imaging and treatment [1,5,6,7,8]. Due to their magnetic properties, IONPs can be directed by external magnetic fields to specific sites in the organism, and thus can be used for the targeted delivery of biotherapeutic agents [7,8,9]. In addition, they can generate heat under the influence of alternating magnetic fields, and thus be used in cancer therapy. [10,11]. In connection with biomedical applications, various types of IONPs with different coatings are available, e.g., the dextran-coated Endorem® or very small superparamagnetic IONPs (VSOPs) [6,12]. VSOPs are citrate coated and have a negative surface charge. Their diameter is about 11 nm and they have a 5 nm iron oxide core. These properties allow for the efficient uptake of the particles into the cells without the need for an additional transfection agent [13]. The small particle size is also intended to reduce possible negative effects on the functionality of MSCs compared to other IONPs [14]. Furthermore, VSOPs appear to have a high biocompatibility. They are biodegradable and are metabolized by the cells in iron metabolic pathways [3,14], although negative effects of degradation products cannot be excluded [14]. Labelling with VSOPs leads to a significant reduction of the T2 relaxation time, which, in T_2_*-weighted images, leads to hypointensity, i.e., signal darkening [13,15,16]. Therefore, these particles can be called “negative” MRI contrast agents [17]. The presence of VSOP-labelled cells is shown by a signal loss and hypointense spots in the MRI images [3,14]. 

Different cell types, such as human mesenchymal stem cells (MSCs) from bone marrow (hBMSCs) [14], human adult neural stem cells (haNCSs) [3], murine embryonic stem cells (mESCs) [3] and human umbilical blood mononuclear cells (hUCBC) [16] were labelled with VSOPs and analyzed with respect to particle uptake and detectability by MRI. There are considerable differences between the cell types with respect to particle uptake and labelling efficacy. For example, hUCBC could not achieve the labelling efficacy of mESCs, even using higher VSOPs concentrations. This observation is probably due to the larger cell diameter of mESCs (18–20 μm) with a higher probability of surface dependent endocytotic events [16]. Groups using MSCs as a cell source confirmed reliable and efficient labelling of MSCs with VSOPs [13,14].

The wide range of applications of IONPs leads to an increasing human exposure [18]. However, the knowledge about their interactions with living cells is still incomplete. Due to their physicochemical properties, nanomaterials can potentially induce cyto- and genotoxic effects in human cells, as well as impairment of important cell functions [19]. Influence on the integrity of cell components, such as DNA or mitochondria, can affect cell survival and cause DNA damage or inflammation. There is evidence for the induction of oxidative stress and DNA breakage, impairment of mitochondrial activity, damage to the cell membrane and promotion of apoptosis in cells exposed to IONPs [20]. Consequently, it is imperative to evaluate the dose response relationships between human cell systems and IONPs, as detailed information on the interactions is required. Specifically, with regard to stem cell labelling for long-term in vivo monitoring, there is little data in the long-term effects of cell labelling with IONPs on survival and the specific properties of stem cells, such as their ability to multidifferentiate and communicate with other cell systems via cytokines. 

Long-term in vivo monitoring to assess the efficacy of cell therapy may be mandatory in regenerative medicine and tissue engineering. The tracking of the transplanted cells should be accompanied by functional studies to control the success and safety of cell therapy approaches. Adipose tissue-derived stromal cells (hASCs) are a subgroup of mesenchymal stem cells (hMSCs), and can be obtained in large quantities with low donor site morbidity [21]. They have the ability to differentiate into various mesenchymal tissues such as cartilage [22,23,24,25] and fatty tissue [26]. In addition, ASCs are used to prevent scarring [25,27] promote tissue regeneration [28], due to the secretion of various growth factors and cytokines [24,25,27,28]. In addition, the combination of ASCs with adipose tissue, so-called “cell-assisted lipotransfer” (CAL), is expected to improve long-term volume stability and survival of fat grafts in reconstructive surgery [29,30]. Hence, ASCs are an interesting cell source for stem cell therapy in regenerative medicine. However, labelling agents to track ASCs should not affect the safety of hASCs or their characteristic properties.

Therefore, the present study was conducted to analyze the effect of VSOP labelling on hASCs by including cellular particle distribution, differentiation capacity and cytokine secretion in order to monitor the success of stem cell therapy approaches.

## 2. Material and Methods

### 2.1. Characterization of VSOPs

VSOPs C200 were purchased from Ferropharm (Teltow, Germany). They have a diameter of about 11 nm, a 5 nm iron oxide core and a negative surface charge due to the citrate coating. Information about R1 (22.5 mmol^−1^ s^−1^ at 0.47 T) and R2 (49.7 mmol^−1^ s^−1^ at 0.47 T) relaxivities of VSOP C200 could be found in the publication of Stroh et al. [15]. For labelling of the cells no additional transfection agent is necessary [3,14,17].

Transmission electron microscopy (TEM) was used to determine the morphology and intracellular distribution of the VSOPs. The samples were prepared by drop coating on carbon-coated copper grids after sonication and stabilization. A transmission electron microscope (EM 900, Carl Zeiss, Oberkochen, Germany) was used for evaluation. The investigations were kindly supported and performed by the group of Prof. Dr. Krohne, Division of Electron Microscopy Theodor-Boveri-Institute, University of Wuerzburg.

Dynamic light scattering (Malvern Instruments Ltd., Herrenberg, Germany) was used to determine the size distribution of the VSOPs in the expansion medium. The surface zeta-potential of the dispersion in the expansion medium (pH 7.4) was determined with a ZetaSizer 3000HSA (Malvern Instruments Ltd.). These investigations were kindly carried out by Mrs. Susanne Koch of the ISC Fraunhofer Institute Wuerzburg.

### 2.2. Isolation and Expansion of hASCs

The investigations followed the guidelines of the institutional Ethics Board (#72/06). Informed consent was approved from 6 healthy donors, who underwent liposuction surgery for aesthetical reasons. 

The hASCs were isolated as described previously [31]. In short, the liposuction material was digested with Collagenase P (Roche Diagnostics, Mannheim, Germany) under sterile conditions for three hours with constant shaking. The tissue was then centrifuged, and the supernatant discarded. A lysis buffer [31] was added to eliminate the erythrocytes. After 10 min, a further washing step with phosphate-buffered saline solution (PBS; Roche Diagnostics, Mannheim, Germany) plus 1% penicillin/streptomycin (P/S; Biochrom AG, Berlin, Germany) and a centrifugation step followed. As the expansion medium (EM-DMEM), the Dulbecco’s modified Eagle’s medium (DMEM; Gibco Invitrogen, Karlsruhe, Germany) with 1% P/S and 10% fetal calf serum (FCS; Linaris, Wertheim-Bettingen, Germany) was used. The obtained cell pellet was resuspended in EM-DMEM and maintained at 37°C in a humidified atmosphere and at 5% CO_2_ in culture flasks. Once the cells (passage 0) had reached 80% confluence, they were detached with 0.25% trypsin/1mM EDTA (Gibco Invitrogen) and freezed in cryopreservation medium containing 80% FCS, 10% DMEM and 10% dimethylsulfoxide [DMSO]. For the following analyses, the hASCs were expanded in EM-DMEM under the above-mentioned conditions. The human ASCs of passage 2 from all six patients were used for the analyses. 

### 2.3. Labelling of hASCs with VSOPs

The labelling procedure was carried out according to the manufacturer’s instructions. VSOPs were added to the expansion medium at a concentration of 1.5 mM, followed by 90 min incubation at 37 °C and 5% CO_2_. To remove the remaining extracellular VSOPs, the labelled cells were intensively washed with 1× PBS. Some 24 h after labelling, the cells were detached and used for the analyses. 

### 2.4. Detection and Quantification of VSOPs-Labelled hASCs with TEM and Prussian Blue Staining 

In order to quantify VSOPs-containing cells in culture and to determine the intracellular distribution of VSOPs, TEM analysis was performed, as described previously [32,33,34]. VSOPs-labelled and unlabelled hASCs from three patients were used as pellets 24 h after the labelling procedure and after 7, 14, 21 and 28 days for these analyses. The pellets were fixed with a 0.1 M sodium cacodylate buffer (pH 7.2), plus 2.5% glutaraldehyde and 2% formaldehyde. For post-fixation of the cell pellets, 2% osmium tetroxide in 50 mM sodium cacodylate buffer (pH 7.2) was used. Afterwards, a staining with 0.5% aqueous uranyl acetate was performed. After dehydration and embedding, the samples in epoxy resin (Epon 812) were cut into 60 nm sections. Imaging was performed with a Zeiss transmission electron microscope EM 900 (Carl Zeiss AG). The photographs were digitized by scanning. For each point in time, 20 cells were counted, and the value of unlabelled cells, which did not contain nanoparticles, was put into relation to the labelled cells.

Additionally, a Prussian blue staining of monolayer cultures for intracellular iron detection was performed. Human ASCs were seeded to slides and allowed to adhere overnight 24 h after the labelling procedure and after 7, 14, 21 and 28 days. After fixation of the slides and a washing step, incubation with 1% potassium ferrocyanide in 1% hydrochloric acid and counterstaining with nuclear fast red, followed [35]. The histological images were microscopically recorded and qualitatively analyzed using an inverted Leica DMI 4000B microscope (Leica Microsystems CMS GmbH, Wetzlar, Germany). The cell doubling time was determined after counting the hASCs [14].

### 2.5. Cytotoxicity

The MTT [3-(4,5-dimethylthiazol-2-yl)-2,5-diphenyl tetrazolium bromide] assay [36] was used to detect possible cytotoxic effects and effects of VSOPs-labelling on the proliferation of hASCs. Unlabelled hASCs served as negative controls. Human ASCs treated with 0.1 mM tert-butylhydroperoxide (t-BHP; Luperox® TBH70X; Sigma-Aldrich), which induces cell apoptosis [37], were used as positive controls. For each patient, 8 wells were seeded at each time point and used to calculate mean extinction values. The analyses were performed 24 h after the labelling procedure and after 7, 14, 21 and 28 days. After removal of the medium from each well, 100 µL MTT were added. After an incubation step at 37 °C in a 5% CO_2_ atmosphere for 4 h and removal of the MTT solution, 100 µl isopropanol were added. Thirty minutes later, the color conversion at 570 nm was determined with a Titertek Multiscan PLUS (MKII) multiplate reader (Pforzheim, Germany). The mean extinction values were calculated from 8 wells per patient. The values of the unlabelled hASCs were adjusted to a viability of 100% (viability of untreated ASCs). Viability of the labelled hASCs and the positive controls was presented as a percentage of the viability of the unlabelled hASCs.

### 2.6. Multidifferentiation Capacity

Four weeks after the labelling procedure (day 28), hASCs from passage 6 of all 6 patients were used to evaluate the multilineage differentiation potential after VSOPs-labelling. The adipogenic, chondrogenic and osteogenic differentiation assays were performed as described previously [38]. According to the protocol of Pittenger [39] and modified by Nöth et al. [40], EM-DMEM supplemented with 1 µg/mL insulin and 10 µM dexamethasone, 100 µM indomethacin, as well as 500 µM 1-methyl-3-isobutylxanthine, was used for adipogenic differentiation. For osteogenic differentiation, EM-DMEM with 100 nM dexamethasone, 10 mM ß-glycerophosphate and 50 µg/mL ascorbic acid according to Jaiswal et al. was used [41]. Chondrogenic differentiation was induced with DMEM, plus 1% P/S and 100 nM dexamethasone, 100 µg/mL sodium pyruvate, 50 µg/mL ascorbate-2-phosphate, 40 µg/mL proline, ITS-plus (Sigma-Aldrich), as well as 10 ng/mL TGF-ß3 (LONZA, Basel, Switzerland). Human ASCs cultured in EM-DMEM were used as negative controls. 

The differentiation media were replaced every two days for three weeks. The differentiation capacity was documented by histological images and quantified by Real-Time Polymerase Chain Reaction (PCR). 

#### 2.6.1. Histology

2 × 10^4^ VSOPs-labelled hASCs and unlabelled control cells per well were plated in 4 wells (Greiner Bio-One GmbH, Frickenhausen, Germany). After adipogenic differentiation, intracellular lipid droplets were detected by Oil Red O staining. Extracellular calcium deposits after osteogenic differentiation were confirmed by the presentation of black nodules with the von Kossa stain and red nodules with the Alizarin Red solution. Histology was performed for adipogenic and osteogenic differentiation.

#### 2.6.2. Real Time-PCR Analyses

A total of 1 × 10^5^ VSOPs-labelled hASCs and unlabelled controls per well were seeded in 6 wells (Greiner Bio-One GmbH) and cultured for three weeks in the above-mentioned differentiation media. The hASCs of 6 patients were used for the PCR analyses. Gene expression of adipogenic, osteogenic and chondrogenic marker genes was quantified by Real-Time PCR analyses. Total RNA was extracted with the RNeasy Mini Kit (Qiagen, Hilden, Germany). Reverse transcription was performed with the High Capacity RNA-to-cDNA Master Mix (Applied Biosystems, Darmstadt, Germany) and the Real-time PCR device from Applied Biosystems. Taqman® assays and protocols using 50 ng cDNA per replicate were used for the experiments. To confirm adipogenic differentiation, gene expression of fatty acid binding protein 4 (aP2; NM_001442.2), lipoproteinlipase (LPL; NM_000237.2) and leptin (NM_002303.5) was measured. Alkaline phosphatase (ALP; NM_000478.4), bone gamma-carboxylglutamate protein (BGLAP, osteocalcin; NM_199173.4) and Runt-related transcription factor 2 (Runx-2/cbfa-1; NM_004348.3) are osteogenic markers. Aggrecan (NM_001135.3), the transcription factor SOX-9 (NM_000337.1), COMP (cartilage oligomeric protein, NM_000095.2) and collagen II (COL2A1; NM_033150.2) were used as cartilage marker genes. Relative quantification values (∆CT values) were normalized to the gene expression of GAPDH (NM_002046.3). The values of unlabelled and VSOPs-labelled hASCs were normalized to the gene expression of undifferentiated labelled and unlabelled hASCs (∆∆CT values) and compared.

### 2.7. Expression of Interleukin (IL)-6, IL-8, Vascular Endothelial Growth Factor (VEGF) A and Caspase 3

Real Time PCR analyses were also used to quantify the expression of caspase 3 (CASP3; NM_004346.3), interleukin (IL-) 6 (NM_000600.3), IL-8 (NM_00584.3) and VEGF A (NM_001025366.2). VSOPs-labelled and unlabelled hASCs were harvested 24 h after the labelling procedure and after 7, 14, 21 and 28 days. Relative quantification analyses were performed (∆CT values) normalized to GAPDH.

### 2.8. Statistical Analyses

GraphPad 5 (Graphpad Software, La Jolla, CA, USA) was used for the statistical analyses and graphs. A two-way ANOVA with Bonferroni post tests was used for the MTT assay results and the gene expression values of IL-6, IL-8, VEGF and caspase 3. The unpaired t-test was used for comparative analyses of adipogenic and osteogenic marker gene expression values of VSOPs-labelled and unlabelled controls when the Gaussian distribution could be confirmed. Otherwise, the Mann-Whitney U-Test was used. Significance was assumed for *p* < 0.05 and indicated in the figures by asterisks. The box of the boxplots shows the median, the 1st quartile and the 3rd quartile, and the whiskers depict the minimal and maximal values. The columns show the mean and standard error of the mean (SEM).

## 3. Results

### 3.1. Characterization of VSOPs

**Figure 1:**
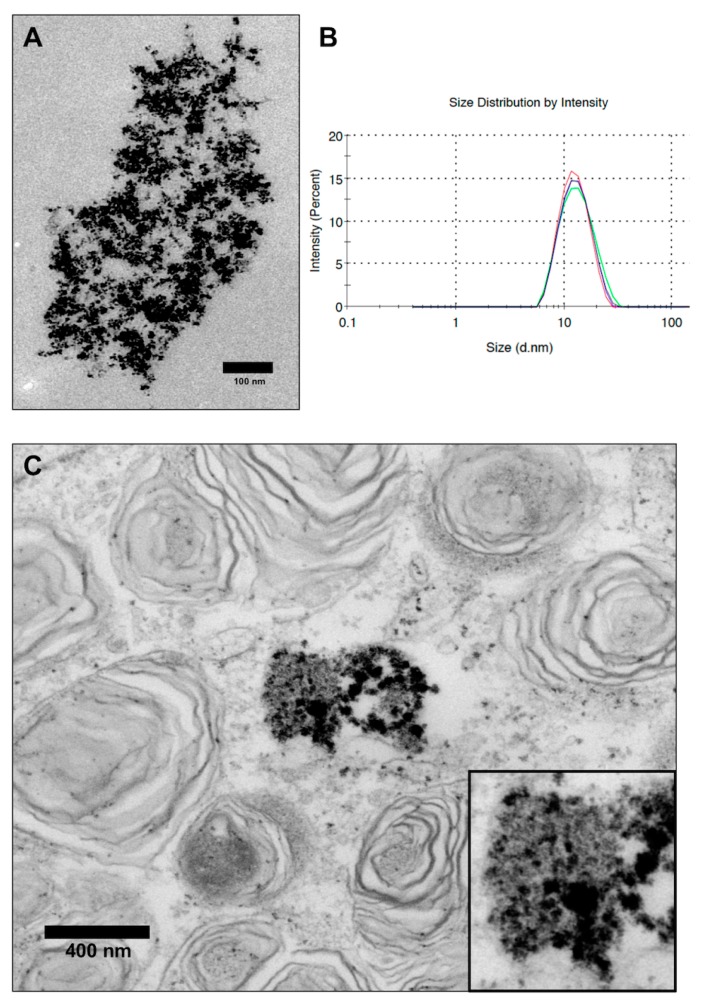
VSOP morphology, characterization and intracellular accumulation: (**A**): Transmission Electron Microscopy (TEM) image of VSOP C200. (**B**): Size distribution diagram of VSOP C200 determined by dynamic light scattering. (**C**): TEM image of intravesicular accumulation of VSOPs in the cytoplasm. The insert shows the intravesicular particles at a higher magnification. The scale bars represent 100 nm (**A)** and 400 nm (**B**) respectively.

According to the manufacturer, the NP-size ranges between 8 and 11 nm. Particles are roundly shaped (Figure 1A). Dynamic light scattering yielded a z-average hydrodynamical diameter of 16.47 nm and a polydispersity index of 0.282 (Figure 1B). The zeta potential at pH 7.4 was −28 mV, with an isoelectric point of 3.2. Intravesicular accumulation of VSOPs was observed when analyzing the TEM images (Figure 1C).

### 3.2. Detection of VSOPs-Labelled hASCs and Quantification during Expansion

**Figure 2:**
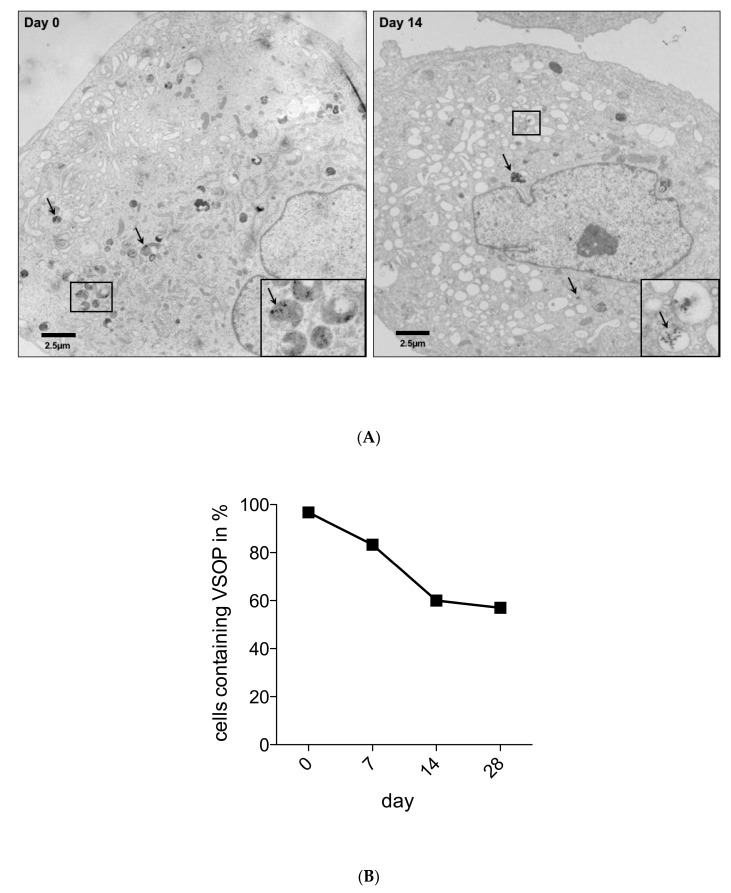
**A:** TEM image analysis: A: Intracellular distribution of intravesicular VSOPs in human ASCs immediately after (day 0) and two weeks (day 14) after labelling. The black arrows show examples of vesicles containing conglomerates of VSOPs (scale bar represents 2.5 μm). The inserts with higher magnification of individual vesicles illustrate the aggregation and deposition of VSOPs in the cytoplasmatic vesicles. **B:** Distribution pattern retrieved by TEM image analysis: A total of 20 cells from three patients were counted for each time point in time and the value of unlabelled cells was compared to the labelled hASCs. Analysis of the TEM images over time revealed a decrease in the percentage of labelled hASCs.

The TEM images show intracellular endosomal vesicles with small iron oxide particles in the labelled hASCs immediately after and two weeks after the labelling procedure (Figure 2A). A total of 20 cells from three patients were counted at each time point, and the value of unlabelled cells was compared to the labelled hASCs. A decrease of the percentage of labelled hASCs during passaging was determined by TEM image analysis (Figure 2B).

**Figure 3:**
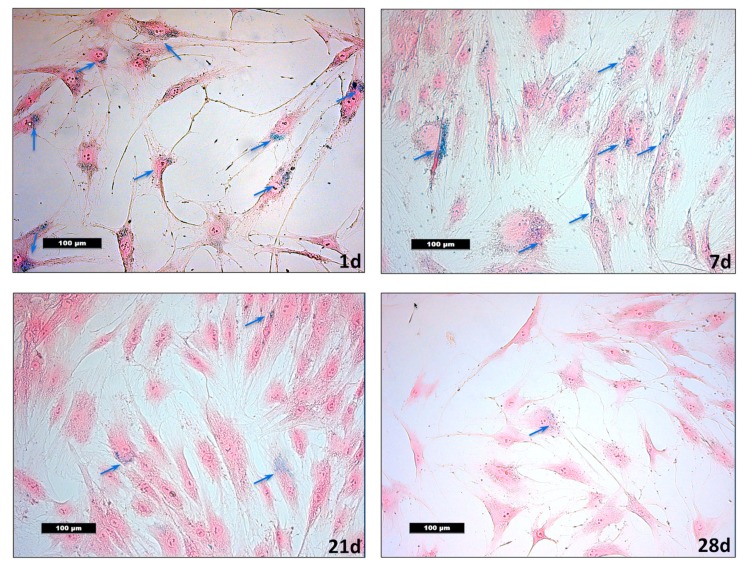
Intracellular very small iron oxide nanoparticles (VSOPs) become visible with the help of the Prussian blue staining. During passage, the percentage of labelled human adipose tissue derived stromal cells (hASCs) seems to decrease compared to unlabelled cells. The histologic images of hASCs 24 h, 7 days, 21 days and 28 days after labelling are shown. Intracellular blue particles are marked by blue arrows. The population doubling time was 5.4 days. Magnification ×200 in all figures; the scale bars represent 100 µm.

Prussian blue staining was performed to confirm the efficient labelling with VSOPs. Immediately after the marking procedure, clear intracellular blue spots could be detected (Figure 3). However, the number of labelled cells decreases during the passage of the hASCs. In the histological images, unlabelled hASCs were increasingly detected over time. The population doubling time was 5.4 days.

### 3.3. Cytotoxicity

**Figure 4:**
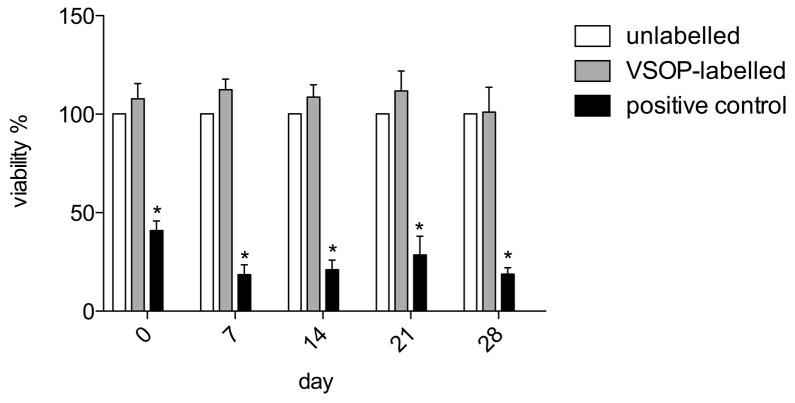
The MTT test was used to assess the influence of the labelling procedure on the proliferation of hASCs and cytotoxic effects. There was no difference between the viability of VSOPs-labelled (light grey columns) and unlabelled cells (white columns) 24 h, 7 days, 14 days, 21 days and 28 days after the labelling procedure. Mean extinction values were averaged from 8 wells per patient and group and normalized to the respective values of unlabelled hASCs from the same patient. The value of the unlabelled hASCs was normalized to a viability of 100% per patient. Human ASCs treated with Luperox® served as positive controls (black columns) and showed a significant decrease in cell viability compared to labelled and unlabelled cells. Significance is indicated by asterisks (*p < 0.001).

The viability of the labelled hASCs was not affected compared to the unlabelled hASCs 24 h and 7, 14, 21 and 28 days after the labelling procedure (Figure 4).

### 3.4. Differentiation Capacity

#### 3.4.1. Histology

**Figure 5:**
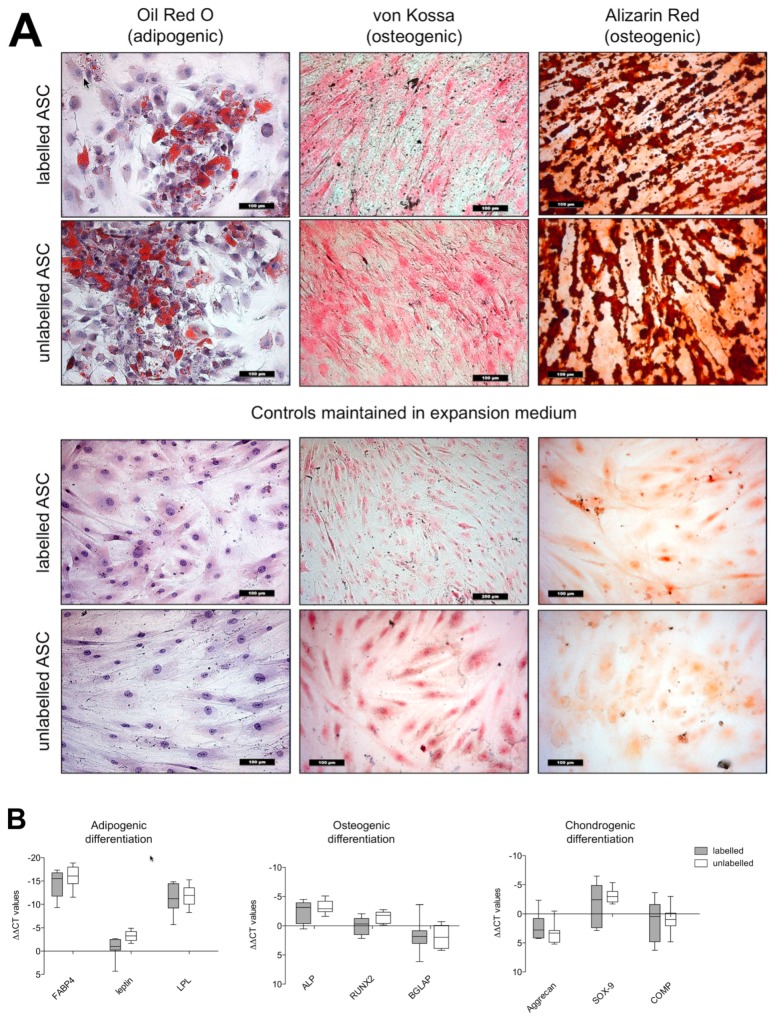
(**A**): Histological results of adipogenic and osteogenic differentiation of labelled and unlabelled hASCs: Oil Red O-stained intracellular lipid droplets could be detected in both labelled and unlabelled cells after two weeks of adipogenic induction (first column). The deposition of calcified extracellular matrix was detected in VSOPs-labelled and unlabelled cells using von Kossa (second column) and Alizarin Red (third column) staining after osteogenic differentiation. There was no noticeable difference in both groups. Labelled and unlabelled hASCs, maintained in an expansion medium, showed neither intracellular lipid vacuoles nor extracellular calcium deposits (row 3 and 4). Magnification ×200 in all figures; scale bars represent 100 µm. (**B**) Real-time Polymerase Chain Reaction (PCR) analyses of the multidifferentiation potential: The gene expression of specific markers was determined after adipogenic, osteogenic and chondrogenic differentiation by VSOPs-labelled and unlabelled hASCs. Relative quantification was performed and presented as values (∆∆CT values), normalized to the gene expression of the housekeeping gene GAPDH and the gene expression of undifferentiated hASCs. VSOP-labelled and unlabelled hASCs showed no differences in the expression of FABP4, leptin and LPL after adipogenic induction. In addition, the expression of the osteogenic marker genes ALP, RUNX2 and BGLAP, as well as the chondrogenic marker genes aggrecan, SOX-9 and COMP, did not differ in both groups. Box-Whisker plots show median, 1st quartile, 3rd quartile as well as minimal and maximal values of ∆∆CT.

The histologic images show no difference in the adipogenic and osteogenic differentiation ability of VSOPs-labelled and unlabelled hASCs. Typical intracellular lipid droplets were detected in both groups (Figure 5A). Labelled and unlabelled ASCs cultured in EM-DMEM did not show intracellular lipid vacuoles. Deposition of extracellular calcium was detected in the VSOPs-labelled and unlabelled cells with Alizarin Red and von Kossa staining (Figure 5A). The negative controls showed no calcium deposition.

#### 3.4.2. Real Time-PCR Analyses

VSOPs-labelled and unlabelled hASCs showed no difference in the gene expression values of adipogenic marker genes such as FABP4, leptin and lipoproteinlipase (LPL). The gene expression of alkaline phosphatase (ALP), RUNX-2 and osteocalcin (BGLAP) was almost identical in the labelled and unlabelled hASCs. Furthermore, the gene expression of the chondrogenic markers did not differ in the two groups (Figure 5B), while collagen II was not expressed and “undetermined” in the PCR analyses in both the labelled and unlabelled group after three weeks of chondrogenic differentiation.

### 3.5. Gene Expression of IL-6, IL-8, VEGF A and Caspase 3

**Figure 6:**
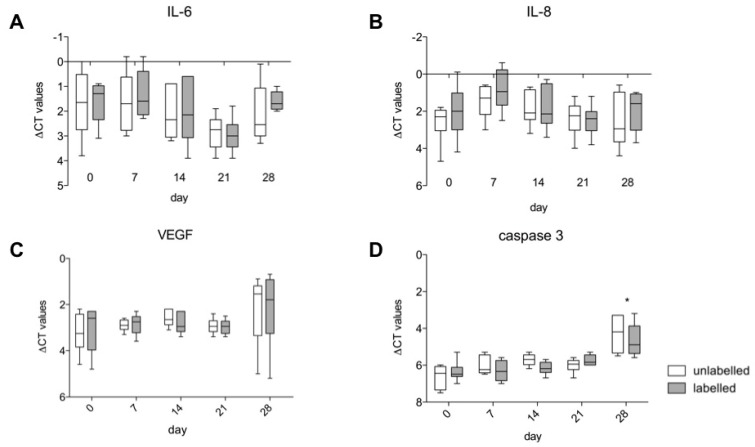
Real-time PCR analysis was used to measure the gene expression levels of IL-6 (**A**), IL-8 (**B**), VEGF (**C**) and caspase 3 (**D**). There were no differences in the gene expression of the VSOPs-labelled and unlabelled hASCs. However, there was a significant increase in the gene expression of caspase 3 after 28 days of culture. The box-whisker plots show the median, the 1st quartile, the 3rd quartile and the minimum and maximum values from ∆CT. Significance is indicated by asterisks (*p < 0.05).

Labelled hASCs and untreated controls showed no differences in gene expression levels (Figure 6A–D).

## 4. Discussion

Iron oxide nanoparticles (IONPs) are increasingly used for various applications, e.g., for the targeted administration of drugs or genes, for cancer imaging and therapy, for hyperthermia, magnetic particle imaging (MPI) and tissue engineering [1,4,5,6,7,9,10,11,42]. In addition, the labelling of MSCs with e.g., citrate-coated VSOPs, allows non-invasive visualization and in vivo tracking by MRI in cellular therapy [1,2,3,14]. This broad use of IONPs can increase human exposure and lead to bioaccumulation of NPs, not only in the target tissue but also in various organs of the recipient [31]. Furthermore, IONPs used for cell labelling in regenerative medicine can have a relevant influence on the physiology and biological behavior of the transplanted cells, and thus influence the success of stem cell treatment.

ASCs are an interesting cell source for stem cell therapy and research. In addition to their ability for multi-lineage differentiation [22,23,24,25,26], ASCs are used in reconstructive surgery to prevent scarring [25,27] and to improve tissue regeneration and graft stability [28,29,30], due to their secretion of various growth factors and cytokines [24,25,27,28].

Information on the influence of IONPs exposition and their intracellular accumulation on the functional capabilities of hASCs is still incomplete and partially controversial [14,43,44]. Our study group previously demonstrated the intracellular accumulation and persistence of ZnO-NPs in hASCs after long-term cultivation [34,45]. Exposure of ZnO-NPs affected cell migration of hASCs [46], but not their ability to multidifferentiate [34,46]. The present study was conducted to add information on the effect of citrate-coated VSOPs on hASCs.

Citrate-coated VSOPs are incorporated into cells by endocytosis and deposited as aggregates in cytoplasmatic vesicles [3]. It could even be seen in Figure 1C and 2A from TEM imaging. Labelling with VSOPs is probably very efficient, due to the diameter of the NPs and the negative surface charge [3]. However, the effectiveness of this seems to depend on the cell size, since cells with a larger diameter such as mESCs show a higher uptake of VSOPs. This could be due to the higher probability of surface-dependent endocytotic events in larger cells [16]. In addition, cells also show a different reduction of T2 relaxation time, which may be a predictor for MRI contrast change after cell transplantation, and allows the estimation of cellular iron oxide uptake [16]. The reduction in relaxation time ranges from 10% [3] to 34% [16] of the control values when analysing mESCs, while other cell types such as mBMCs or hUCBCs required much higher concentrations of VSOPs, but did not reach the T2 relaxation time of mESCs [16]. The detectability of cells also depends on the protocols for MRI adjustment: In the literature, for example, detection limits of 100 ESCs at 17.6 Tesla [13] or 5 × 10^4^ haNSCs at 3 Tesla have been published [3]. Therefore, uniform statements on the detectability of VSOP-labelled cells by MRI are not possible.

In the present study, an even intravesicular accumulation of VSOPs in the cytoplasm of hASCs was found after the labelling procedure by TEM image analysis. Additionally, small blue intracellular spots were detected by Prussian blue staining as signs of incorporated VSOPs. However, cultivation of the hASCs over four weeks resulted in a reduced percentage of labelled cells. Quantification of the labelled cells compared to unlabelled cells over time was also obtained by analysis of TEM images. While other authors also noted a significant reduction in labelled hBMSCs over several passages in the monolayer culture [14], these results are in contrast to those of others who described stable labelling of human adult neural stem cells (haNCSs) over 28 days using the same particles [3]. This may be due to the different cell types used in the experiments, although both hASCs and haNCSs, are proliferating cells. However, it is known that the intracellular concentration of the labelling substance decreases during proliferation. This is partly due to the uptake of the particles by the daughter cells, and partly due to the degradation of the particles via iron metabolic pathways [3,14,38]. This may lead to a short detectability of the labelled cells in the MRI, which could limit long-term observations over months [3]. Little is known about the mechanisms of particle exclusion from the cells. The cell culture conditions do not provide the opportunity to sufficiently investigate such questions. The biodistribution of particles and its consequences are not yet well understood and should be a major topic of future nanotoxicological assessments [18].

Viability of hASCs and their gene expression of IL-6, IL-8, VEGF and caspase 3 were measured weekly over 4 weeks after labelling with 1.5 mM VSOPs. In addition, the potential effects of the VSOPs on the multilineage differentiation ability of hASCs 4 weeks after labelling were evaluated, based on visual observations of histological images and quantitative analysis of adipogenic, osteogenic and chondrogenic marker gene expression. The MTT assay did not confirm any effects on the viability of the hASCs 24 h after the labelling procedure, and after 7, 14, 21 and 28 days. Furthermore, in contrast to the results of others [47], no induction of caspase 3 gene expression, an important factor in the apoptotic pathway of the cells, was determined by PCR analysis. Stroh et al., who used the same concentration of the same IONPs, also found no effect on the cell viability of haNSCs and murine embryonic stem cells (mESC) 8 h and 48 h after the labelling procedure [3]. Some authors describe a concentration-dependent impairment of the viability of ASCs, BMSCs and other cell types after labelling or exposure to IONPs [3,15,47,48], which is thought to be mediated by various cellular changes such as oxidative stress [3,15,42,44,47,49].

Concentration-dependent limiting effects of IONPS have also been observed on the differentiation ability of stem cells [44,49,50]. In the present study, labelled hASCs underwent adipogenic, chondrogenic and osteogenic differentiation for three weeks, and no clear differences between labelled hASCs and unlabelled cells were found in the histological images after adipogenic and osteogenic differentiation. In addition, no differences in specific marker gene expression for fat, bone and cartilage tissue were detected. Collagen II was not expressed by both labelled and unlabelled cells, so that an evaluation of the differences in collagen II expression between the two groups is not possible. These results indicate that VSOPs-labelled hASCs can be expanded without affecting their viability and their ability to differentiate. This is consistent with the findings of others, who have not described any influence of IONPs on the differentiation capacity of different cell types, such as bone marrow-derived stem cells (BMSCs) [51,52], haNCSs [3] or ASCs [53].

Gene expression of IL-6, IL-8 and VEGF showed no difference between labelled hASCs and untreated controls. IL-6 and IL-8 are chemotactic cytokines and mediators of stem cell activation [54]. Furthermore, MSCs promote tumor cell progression via IL-6 secretion and proangiogenic factors [55]. In contrast to the present study, other metal oxide nanoparticles, such as silver oxide NPs [32] and zinc oxide NPs [56], lead to the increased secretion of those cytokines.

In summary, under conditions similar to those of labelling procedures in regenerative medicine and tissue engineering, we do not assume a relevant cytotoxic potential or adverse effects of VSOPs on hASCs. Although no acute or subacute effects on cellular viability or functionality have been observed, particle accumulation in the spleen or liver and organ toxicity under in vivo conditions must be considered. Therefore, future experiments should address the biodistribution of VSOPs as used in drug delivery, magnetic hyperthermia, cancer therapy and magnetic particle imaging studies.
